# Development of a PCR-based technique for genotyping UGT1A1 gene and distribution of rs3064744 alleles in the Russian population

**DOI:** 10.3389/fgene.2026.1899437

**Published:** 2026-08-26

**Authors:** M. A. Vinokurov, K. O. Mironov, M. S. Yurchuk, V. G. Akimkin

**Affiliations:** Central Research Institute for Epidemiology, Federal Service for Surveillance on Consumer Rights Protection and Human Wellbeing, Moscow, Russia

**Keywords:** 1D-CNN, deep learning, Gilbert’s syndrome, melting curve analysis (MCA), pharmacogenetics, RS3064744, TA-repeats, UGT1A1

## Abstract

**Background:**

Accurate determination of tandem thymine-adenine (TA) repeat numbers in the UGT1A1 promoter region (rs3064744) is essential for diagnosing Gilbert’s syndrome and personalizing therapy with toxic agents like irinotecan and atazanavir. However, traditional polymerase chain reaction (PCR) assays face severe limitations due to the AT-rich sequence and overlapping melting temperatures (Tm) of the highly homologous 7TA and 8TA alleles. In this context, melting curve analysis (MCA) employing fluorophore-quencher systems has emerged as a promising alternative. The purpose of this study was to develop a novel genotyping approach combining optimized aPCR-MCA analysis with an automated classifier to overcome the limitations posed by the differentiation of highly homologous alleles and to demonstrate its practical application, providing the distribution of rs3064744 genotypes across four regional cohorts of the Russian population.

**Methods:**

A specialized Dual Head 1D-convolutional neural network (1D-CNN) ensemble with Test-Time Augmentation (TTA) was developed. The model was trained and internally validated on 1,620 engineered plasmid samples, and independently evaluated on an external clinical test set of 440 unique patient genomic DNA specimens. Real-time PCR was performed on CFX96 and DTprime platforms. Additionally, population-wide screening was conducted on 997 archival clinical samples from Moscow, Sakha (Yakutia), Dagestan, and Rostov regions.

**Results:**

While 5TA and 6TA alleles were easily separated, absolute Tm distributions of 7TA and 8TA alleles overlapped significantly, and non-uniform Tm shifts of 0.8 °C–1.4 °C occurred across platforms. Conventional absolute Tm thresholding was therefore inadequate. By assessing relative morphological curve divergence against co-amplified 7TA/7TA and 7TA/8TA reference anchors, the 1D-CNN ensemble neutralized instrument noise. It achieved 100% accuracy on internal validation and 100% concordance (440/440) with clinical reference pyrosequencing. Population screening revealed that Dagestan, Yakutia, and Rostov cohorts closely align with the European population. Rare 5TA and 8TA alleles were detected at low frequencies in Yakutia and Moscow.

**Conclusion:**

Combining LNA-modified aPCR-MCA with a comparative 1D-CNN model successfully circumvents thermodynamic limitations and eliminates human operator bias. This integrated system offers an accessible, high-throughput, and clinically valid solution for routine UGT1A1 pharmacogenetic testing.

## 1 Background

The uridine diphosphate glucuronosyltransferase 1A1 enzyme (UGT1A1) is crucial for hepatic glucuronidation of bilirubin and detoxification of various exogenous substances, including pharmacological agents. The expression of the *UGT1A1* gene is significantly influenced by the number of tandem thymine-adenine (TA) repeats in its core promoter region (TATA-box), a polymorphism designated as rs3064744 (Raijmakers et al., 2000).

The wild-type allele usually contains six repeats and is denoted as UGT1A1*1 (A(TA)_6_TA). Mutant alleles can have five UGT1A1*36 (A(TA)_5_TA), seven UGT1A1*28 (A(TA)_7_TA), or eight UGT1A1*37 (A(TA)_8_TA) repeats (UGT1A1 allele nomenclature, 2015), affecting baseline enzyme expression (Raijmakers et al., 2000). Homozygosity or heterozygosity for alleles associated with reduced enzymatic activity, such as UGT1A1*28 and UGT1A1*37, is the primary genetic factor contributing to Gilbert’s syndrome (GS), a common condition characterized by mild unconjugated hyperbilirubinemia (Du et al., 2019). *UGT1A1* genotyping is used not only in the context of benign hyperbilirubinemia but also in pharmacogenetics. People with the UGT1A1*28 allele have a significantly increased risk of severe adverse reactions, including life-threatening neutropenia, when taking irinotecan, a first-line chemotherapy drug used in oncology (Ibrahim et al., 2024; Loriot et al., 2024; Tuteja et al., 2025). Testing for this allele is also included in the Russian guidelines for the treatment of HIV infection and related diseases, as well as for the chemoprophylaxis of HIV infection when using atazanavir as an antiretroviral drug (Pokrovsky et al., 2025).

The genotyping of the rs3064744 polymorphism presents significant technical challenges. The *UGT1A1* promoter sequence is characterized by a high AT content, which adversely affects the annealing temperature and target specificity of standard TaqMan probes (Daprà et al., 2021). Consequently, traditional real-time polymerase chain reaction (PCR) assays often exhibit decreased discrimination capability. This results in a heightened probability of both false positive and false negative results. To reduce these limitations, alternative diagnostic modalities have been explored, though each carries distinct operational drawbacks. Standard Sanger sequencing, while definitive, remains cost-prohibitive, labor-intensive, and inaccessible for routine practice. Capillary electrophoresis-based fragment analysis is frequently confounded by polymerase slippage and prominent stutter peak artifacts that obscure precise short tandem repeat (STR) allele calling (Sissung et al., 2020). Other PCR-based techniques, such as SYBR Green melting, high-resolution melting, pyrosequencing, and dual-probe systems have expanded the testing landscape, yet achieving unambiguous, low-cost method within a single reaction tube remains a challenge (Sissung et al., 2020). In this context, melting curve analysis (MCA) employing fluorophore-quencher systems has emerged as a promising alternative (Kong et al., 2022), particularly when integrated with asymmetric PCR (aPCR) and locked nucleic acid (LNA) modifications to artificially elevate the melting temperature (Tm) and enhance specificity (You et al., 2006; Johnson et al., 2004).

However, the translation of MCA into a fully automated diagnostic workflow continues to present a significant challenge. The classification of highly homologous alleles, particularly the differentiation between the rare 8TA and 7TA variants, often relies on subjective manual interpretation or rigid heuristic algorithms, which are susceptible to hardware-specific thermal noise and fluorescence baseline shifts (Mergny and Lacroix, 2003). In recent years, the integration of deep learning techniques has emerged as a powerful approach for post-PCR data processing. The approaches have demonstrated significant utility in automating genotype interpretation and enhancing classification robustness by extracting hierarchical features directly from complex melting curve morphologies, thereby reducing operator-dependent bias (Ozkok and Celik, 2022; Promja et al., 2023).

The purpose of this study was to develop a novel genotyping approach combining optimized aPCR-MCA analysis with an automated classifier to overcome the limitations posed by the differentiation of highly homologous alleles and to demonstrate its practical application, providing the distribution of rs3064744 genotypes across four regional cohorts of the Russian population.

## 2 Materials and methods

### 2.1 Samples, DNA extraction, PCR, and sequencing

To investigate the distribution of the rs3064744 allele frequency in the Russian population, a cohort of 997 individuals from four distinct regions was established: Moscow and Moscow Region (n = 371), the Republic of Sakha (Yakutia) (n = 310), the Republic of Dagestan (n = 202), and the Rostov Region (n = 114).

The cohort was constituted using archival genomic DNA samples obtained through retrospective analysis from a collection originally established for an independent epidemiological study. The median age of the subjects at the time of initial sampling was 37 years (range: 18–54 years).

For the development and validation of the machine-learning models, an additional dataset was assembled, comprising melting profiles from 1,620 model samples and 440 clinical genomic DNA samples. The clinical specimens were derived from whole blood of patients tested for *UGT1A1* genotype at the «Center of Molecular Diagnostics» clinic (Moscow, Russia) on suspicion of hyperbilirubinemia, collected over the period of 2014–2026. This diagnostic cohort differs in both purpose and composition from the archival population cohort (n = 997) and is not intended to represent the general Russian population. The samples were taken without any selection or sorting by genotype; however, because testing was prompted by a phenotype associated with reduced *UGT1A1* activity, the cohort over-represents reduced-function genotypes relative to unselected population samples, including rare 8TA-containing combinations that are near-absent at the population level. This clinical-indication bias was advantageous for the present work, as it provided native clinical material with which to challenge the classifier on the genotype boundaries most prone to misclassification. Reference genotyping was performed using the «AmpliSens® Pyroscreen UGT1A1-screen» kit (AmpliSens, Russia), a registered *in vitro* diagnostic medical device that, according to the manufacturer’s instructional manual, has been clinically validated against Sanger sequencing (Dribnokhodova et al., 2014; Federal service for surveillance in healthcare, 2026).

Model samples were generated using plasmid constructs containing a 128–131 base pair fragment of the *UGT1A1* gene, which include the rs3064744. The DNA fragment was inserted into the pGem-T cloning vector via standard molecular cloning techniques (Sambrook et al., 1989). Plasmids carrying the 6TA and 7TA alleles were derived from homozygous clinical specimens. In order to obtain the rare 5TA and 8TA alleles, fragments were cloned from heterozygous clinical samples exhibiting the respective genotypes. The genotypes 5TA/6TA, 5TA/7TA, 5TA/8TA, 6TA/7TA, 6TA/8TA, and 7TA/8TA were recreated by combining these isolated cloned fragments. All cloned controls were measured using real-time PCR. Primers specific to the pGem-T vector sequence were designed, along with calibrator reagents from the Central Research Institute of Epidemiology (Russia) using Rotor-Gene instrument (Qiagen, Germany). The model samples were subsequently diluted to four different concentrations: 5 × 10^5^, 2 × 10^4^, 1 × 10^4^, and 5 × 10^3^ copies per milliliter. This approach ensured a comprehensive presence of all possible allelic permutations in the training dataset.

Genomic DNA was extracted from whole blood using the «RIBO-prep» kit. PCR was conducted using CFX96 (Bio-Rad, United States) and DTprime (DNA-Technology, Russia) instruments.

The reaction mixture was composed of 25 μL, which included 10 μL of the primer-probe-dNTP mix, 4.5 μL of RT-PCR mix-2 FEP/FRT, 0.5 μL of TaqF polymerase, and 10 μL of extracted DNA template. The primer-probe-dNTP mix contained 160 nM (final concentration in the PCR reaction) of the limiting forward primer, 640 nM of the excess BHQ1-labeled reverse primer, and 160 nM of the LNA-modified probe. The total primer concentration was initially optimized across a range of 200–800 nM (tested at 200, 400, 600, and 800 nM, all values referring to the final reaction mixture), with 800 nM determined as optimal to ensure maximal asymmetric amplification efficiency and a dNTP concentration of 0.44 mM. The established primer ratio and absolute concentrations were validated to ensure robust discrimination across all four allele types (5TA, 6TA, 7TA, and 8TA) in a single-tube format. All reagents and kits used for PCR and DNA extraction were manufactured by AmpliSens (Russia). The thermal cycling protocol involved initial denaturation at 95 °C for 15 min, followed by 45 cycles of denaturation at 95 °C for 5 s and annealing/extension at 60 °C for 20 s. The 45-cycle protocol is an intrinsic requirement of the asymmetric PCR design. Following depletion of the limiting forward primer during the exponential phase, the reaction enters the linear amplification phase; the extended cycling is therefore strictly necessary to ensure sufficient accumulation of single-stranded DNA targets for subsequent probe hybridization. Following amplification, melting curve analysis was performed in a gradient from 25 °C to 70 °C with continuous fluorescence detection on the channel for FAM-labeled probe detection.

### 2.2 Bioinformatics analysis of global populations

To analyze the population frequencies of the *UGT1A1* alleles, whole-genome sequencing data from 2,504 unrelated individuals was obtained from the 1000 Genomes Project (Phase three) (1000 Genomes Project, 2026). The sample set encompassed representatives from five major super-populations: African (AFR), European (EUR), East Asian (EAS), South Asian (SAS), and Admixed American (AMR). Targeted extraction (slicing) of reads mapping to the *UGT1A1* locus, which includes the rs3064744 polymorphism, was performed directly from the EBI servers using the samtools utility (version 1.23.1) and aligned to the GRCh38 reference genome. The extracted reads were converted into BAM format. STR length determination was conducted using the HipSTR algorithm (version 0.6.2) (Willems et al., 2017). To improve genotyping accuracy, the built-in polymerase stutter error compensation model (--def-stutter-model) and a minimum read depth filter (--min-reads 10) were applied. The resulting data were merged into a multi-sample Variant Call Format (VCF) file using the mergeSTR tool from the TRTools suite (Mousavi et al., 2021). The final calculation of genotype distributions and allele frequencies across populations was performed using a custom Python script. To establish a comprehensive comparative baseline, aggregated allele frequencies were additionally retrieved from the Genome Aggregation Database (gnomAD) v4.1.1 (European cohort) (Guez et al., 2026) and the Russian national genome repository Federal Medical-Biological Agency (FMBA) Genome Database (Author Anonymous, 2026). For these aggregated databases, the frequency of the wild-type 6TA allele was calculated as the residual percentage (100% minus the sum of frequencies of the minor alleles: 5TA, 7TA, and 8TA). Statistical comparisons of allele and genotype distributions between all cohorts were performed using Pearson’s Chi-square (χ^2^) test of independence. To ensure the statistical validity and to mitigate the potential mathematical biases associated with low expected cell counts, rare alleles (5TA and 8TA), which collectively accounted for less than 1% of the total observations, were excluded from the primary chi-squared calculations.

### 2.3 Machine learning

The principal model for automated genotype interpretation employs a specialized one-dimensional convolutional neural network a Dual Head 1D-CNN model. To ensure complete reproducibility, a rigorous standardization process was implemented for the fluorescence preprocessing pipeline prior to deep learning analysis. The initial fluorescence measurements, comprising 46 data points per sample, were utilized without any arbitrary algorithmic smoothing or manual baseline adjustment. The first and second derivatives of fluorescence with respect to temperature were calculated using second-order accurate central difference methods. To mitigate inter-run baseline shifts, absolute amplitude variations, and hardware-specific scaling, a Z-score normalization technique was applied. The 1D-CNN model employs a convolutional backbone composed of sequential blocks, each containing a «Conv1d» layer, followed by «BatchNorm1d», «ReLU» activation, and «Dropout». Input curves are processed through these layers and subsequent pooling stages («MaxPool1d» and «AdaptiveAvgPool1d») to extract a high-dimensional feature vector of 256 elements. This vector is fed into a shared fully connected layer before being divided into two independent classification heads (Head one and Head 2), each tasked with predicting a single allele (e.g., Allele 1: 6TA, Allele 2: 7TA). To mitigate the risk of overfitting and ensure model robustness, a comprehensive regularization strategy was implemented. A Focal Loss function was utilized in place of standard cross-entropy to dynamically adjust the loss and direct the network’s attention toward challenging-to-classify instances, such as the overlapping thermal profiles of 7TA/7TA and 7TA/8TA genotypes. Additionally, a control rotation technique was applied during training by randomly selecting reference samples (7TA/7TA and 7TA/8TA anchors) from a pool of identical genotypes within each batch. This approach prevents the model from memorizing hardware-specific noise associated with static control curves. Furthermore, Mixup augmentation was employed via linear interpolation between input tensors to create virtual training samples, thereby enhancing model generalization. To maximize diagnostic reliability, an ensemble of five independent models, each initialized with distinct random seeds, was deployed. During inference, Test-Time Augmentation (TTA) was applied by introducing random Gaussian noise to the input curves across five iterations. The final prediction was determined by averaging the resultant class probabilities.

To evaluate the necessity of deep learning, three tree-based algorithms: Random Forest (configured with 200 estimators), XGBoost (utilizing a multiclass log-loss objective), and LightGBM were trained for comparison. For these classical models, the 11-channel input tensor (11 × 46 data points) was flattened into a high-dimensional vector of 506 features. To ensure a fair comparative analysis, these algorithms utilized the identical Dual Control Sample Comparison feature space, incorporating the differential curves relative to the 7TA/7TA and 7TA/8TA anchors.

The machine learning workflow employed a rigorous biological partitioning strategy. The 1,620 engineered plasmid model samples were divided into a training set (n = 1,295) and an internal validation set (n = 325), using a stratified sampling approach with a ratio of 80/20 (Table 1). To ensure unbiased performance evaluation and prevent target leakage, the 440 clinical genomic DNA specimens were completely separated from all stages of model development, serving exclusively as an independent external validation cohort.

**TABLE 1:** Genotype distribution of the plasmid model samples portioned into training and internal validation sets.

Genotype	Total model samples (n = 1,620)	Training set (80%, n = 1,295)	Internal validation set (20%, n = 325)
5TA/5TA	134	107	27
6TA/6TA	134	107	27
7TA/7TA	226	181	45
8TA/8TA	226	181	45
5TA/6TA	134	107	27
5TA/7TA	135	108	27
5TA/8TA	134	107	27
6TA/7TA	134	107	27
6TA/8TA	135	108	27
7TA/8TA	228	182	46

Model performance was evaluated using overall accuracy and the macro-averaged F1-score to assess predictive efficacy evenly across all 11 potential genotype classes. Furthermore, detailed per-class precision and recall metrics were generated to identify specific algorithmic vulnerabilities in differentiating genetically similar alleles.

## 3 Results

### 3.1 aPCR-based way for rs3064744 genotyping

**FIGURE 1:**
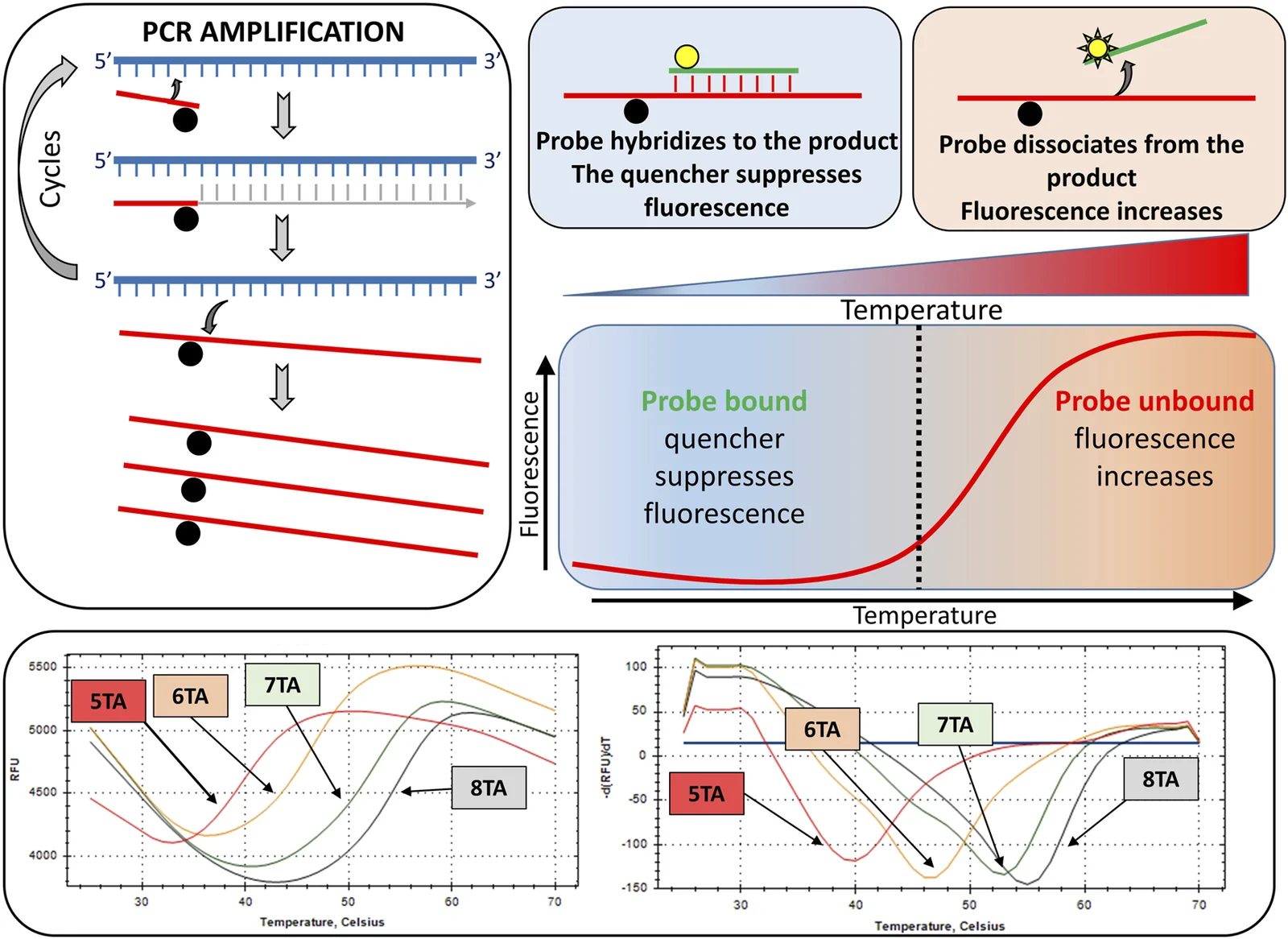
Schematic representation of the MCA methodology.

An alternative methodology was devised, combining aPCR with MCA. The assay consists of three sequential steps within a single reaction tube (see Figure 1):

1. In the first phase, a deliberate disparity in the concentrations of the forward and reverse primers is established. Specifically, the reverse primer, modified with a fluorescence quencher (BHQ1), is present in substantially greater quantities than the unmodified forward primer. During the early cycles of the PCR process, exponential amplification occurs, with both primers available in sufficient amounts for polymerase to synthesize double-stranded DNA products. As thermal cycling progresses, the limiting forward primer becomes completely depleted. Subsequently, polymerase-mediated elongation exclusively occurs from the excess reverse primer. This transition from exponential to linear amplification results in the continuous accumulation of single-stranded DNA targets incorporating BHQ1, which serve as substrates for subsequent probe hybridization.
2. Following thermal cycling, the reaction mixture is cooled to conditions that facilitate specific oligonucleotide annealing. At this point, the predominant target component is a single-stranded amplicon that contains an internal, quencher. A fluorescently labeled probe specifically hybridizes with this modified amplicon, forming a heteroduplex. The spatial arrangement of the system ensures that the probe’s reporter fluorophore and quencher are within an effective interaction for fluorescence quenching.
3. In the final stage, the mixture is subjected to controlled heating while fluorescence intensity is continuously monitored. At the specific Tm of the probe-target complex, the probe dissociates from the amplicon, leading to the spatial separation of the fluorophore and quencher. This dissociation results in a rapid increase in fluorescence intensity. By plotting the negative derivative of fluorescence with respect to temperature (-dF/dT), a distinct bell-shaped peak is generated. The peak’s apex corresponds precisely to the Tm of the specific allele, enabling clear genotype differentiation.

The discriminatory power of the assay is exclusively determined by the structure of the hybridization probes, as primer design was found to exert no influence on analytical specificity. Through the PCR optimization, the following results were obtained:.

1. The optimal concentration ratio of BHQ-labeled (excess) primers to unmodified (limiting) primers was established to be within the range of 3:1 to 4:1. At lower concentrations of BHQ-labeled primers (as 2:1), there is a potential for the occurrence of overlapping peaks from closely related sequences, such as 7TA and 8TA. However, this ratio is suitable for differentiating the most prevalent genotypes, including 6TA/6TA, 6TA/7TA, and 7TA/7TA (see Supplementary Figure S1 for peak separation profiles).
2. The distance between labels within the hybridized duplex is a crucial determinant of signal amplitude. Experimental determination revealed that the optimal distance ranges from 4 to 10 nucleotides (nt). Distances shorter than 4 nt may weaken probe hybridization efficiency, while distances greater than 10 nt may reduce quenching efficiency and increase background noise level.
3. DNA probes exhibit limited efficacy for genotyping the AT-rich regions of the *UGT1A1* gene due to their low thermal stability and reduced specificity. To overcome this thermodynamic barrier, we integrated LNA monomers into the probe sequence. LNA nucleotides feature a modified ribose sugar constrained by a methylene bridge connecting the 2′and 4′positions. This modification introduces a structural rigidity that significantly increases the binding affinity for complementary target sequences. Consequently, the incorporation of LNA optimally elevates the Tm, minimizes unwanted off-target hybridization, and ensures precise discrimination between closely related allelic variants.
4. Experimental data indicated that the optimal number of LNA modifications for robust differentiation of the four alleles (5TA, 6TA, 7TA, and 8TA repeats) ranges from 6 to 10. While increasing the number of LNA modifications enhances specificity, it concurrently increases synthesis complexity and reagent costs (Table 2).

**TABLE 2:** Oligonucleotide sequences.

Name	5′-3′ sequence	Final concentration, nM
Forward	gCT ACC TTT gTg gAC TgA CAg CTT	160
Reverse	CTg CCA gAg gTT CgC CCT CT (BHQ1)C	640
Probe*	TTT gCC AT+A T+AT +ATA TA+T AT+A TA+T +AT(FAM)A Ag+T +Agg A(P)	160

* The «+» before a nucleotide indicates an LNA, monomer.

The melting temperatures of the four alleles, measured separately on each platform, are summarised in Table 3, giving the mean, standard deviation, median, and observed range for every homozygous and heterozygous class. The 5TA and 6TA alleles are separated from one another and from the 7TA/8TA cluster by wide, non-overlapping margins on both instruments, permitting unambiguous assignment by peak position alone.

**TABLE 3:** Experimentally determined Tm for all *UGT1A1* promoter region homozygous and heterozygous genotype classes.

​	CFX96		DTprime	
Genotype	Mean Tm peak 1 ± SD (Min-Max)	Mean Tm peak 2 ± SD (Min-Max)	Mean Tm peak 1 ± SD (Min-Max)	Mean Tm peak 2 ± SD (Min-Max)
5TA/5TA	39.9 ± 0.5 (38.9–40.7)	–	40.6 ± 0.3 (40.3–41.7)	–
6TA/6TA	46.7 ± 0.5 (45.9–47.8)	–	47.6 ± 0.3 (47.2–48.9)	–
7TA/7TA	52.7 ± 0.5 (52.0–54.1)	–	53.8 ± 0.3 (53.2–55.7)	–
8TA/8TA	54.9 ± 0.6 (54.2–56.5)	–	56.3 ± 0.3 (56–57.8)	–
5TA/6TA	40.3 ± 0.7 (38.8–42.0)	46.4 ± 0.7 (45.2–48.2)	40.6 ± 0.3 (40.1–41.8)	47.6 ± 0.3 (47–48.9)
5TA/7TA	40.3 ± 0.6 (38.9–41.6)	52.7 ± 0.6 (51.7–54.2)	40.6 ± 0.3 (40.1–41.8)	53.9 ± 0.4 (53.4–55.4)
5TA/8TA	39.8 ± 0.5 (39.1–41.4)	55.1 ± 0.7 (54.3–56.6)	40.4 ± 0.3 (40–41.7)	56.5 ± 0.4 (56.1–58.3)
6TA/7TA	47.3 ± 0.6 (45.6–48.8)	53 ± 0.8 (51.3–54.8)	47.6 ± 0.3 (47.3–49)	53.8 ± 0.4 (53.5–55.3)
6TA/8TA	47.3 ± 0.5 (46.4–48.8)	54.6 ± 0.6 (53.5–56.4)	47.7 ± 0.3 (47.4–49.1)	56.3 ± 0.3 (55.7–57.9)
7TA/8TA	53.7 ± 0.5 (52.8–55.5)	–	54.4 ± 0.4 (53.9–56.3)	–

The 7TA and 8TA alleles behave differently. Although their homozygote means differ by 2.1 °C on CFX96 (52.7 °C vs. 54.9 °C) and 2.5 °C on DTprime (53.8 °C vs. 56.3 °C), the observed distributions overlap extensively once the full range is considered. On CFX96, individual 7TA/7TA peaks were recorded as high as 54.1 °C and 8TA/8TA peaks as low as 54.2 °C, while the merged 7TA/8TA heterozygote peak spanned 52.8 °C–55.5 °C across the entire interval occupied by both homozygotes. No single Tm value separates the three classes. The ambiguity is compounded across platforms: absolute Tm shifts non-uniformly by 0.8 °C–1.4 °C between instruments, such that a homozygous 7TA/7TA sample on DTprime (up to 55.7 °C) can register a higher Tm than a homozygous 8TA/8TA sample on CFX96 (from 54.2 °C). These differences are moreover comparable to the native thermal resolution of the instruments (0.5 °C for CFX96, 1.0 °C for DTprime; sub-step values were recovered by cubic-spline interpolation of the raw fluorescence).

These data establish that absolute melting temperature is not a reliable criterion for discriminating the 7TA and 8TA allele combinations, within or across platforms. This reflects a limitation of the summary statistic rather than of the signal: the peak apex is a single scalar that discards the remainder of the melting profile, whereas the genotype-discriminating information is distributed across the full curve morphology and its derivatives, as confirmed by the complete concordance of the morphology-based classifier with the independent reference method (Section 3.2). Accordingly, the assay does not apply Tm thresholding to these classes; it interprets the complete melting profile relative to co-amplified reference genotypes.

### 3.2 Development of a machine-learning model for automatic allele discrimination

To automate the interpretation of melting curve data, a specialized 1D-CNN architecture with two classification modules was developed. A defining feature of this solution is the dual control sample comparison mechanism.

These specific reference anchors (7TA/7TA and 7TA/8TA) were deliberately selected because the 7TA and 8TA transition represents the most thermodynamically challenging discrimination boundary in the assay, characterized by physically overlapping melting profiles.

Recognizing that genotype discrimination patterns at this narrow boundary can vary across different PCR runs, incorporating these specific anchors within each batch forces the neural network to evaluate relative morphological divergence, enabling the algorithm to learn the relative positioning of the curves rather than relying on absolute fluorescence values.

**FIGURE 2:**
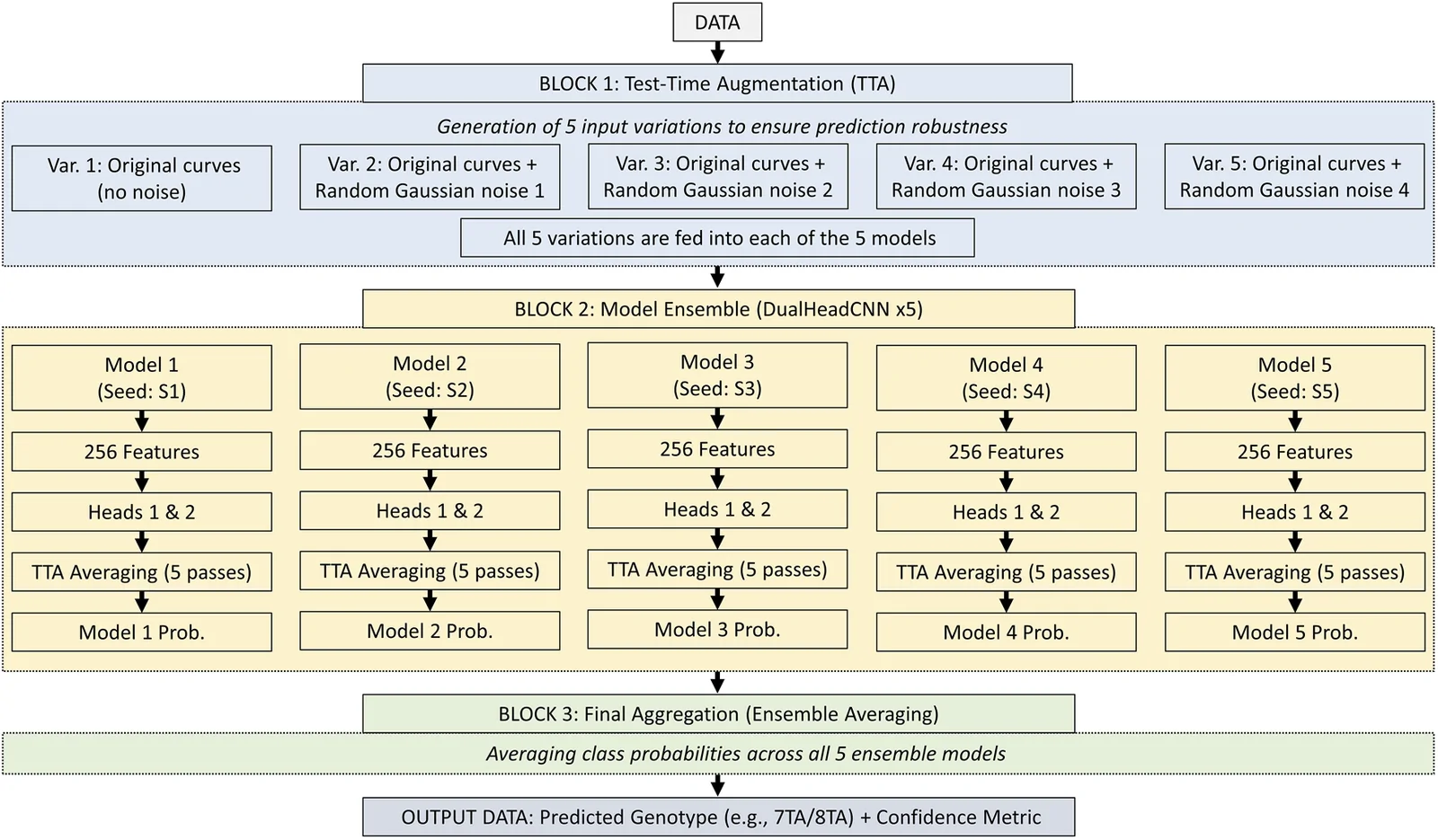
Schematic representation of the 1D-CNN architecture.

The model processes a tensor containing 11 informational channels. These channels include the normalized original signal for each sample, as well as its first and second derivatives. For each reference, the input encompasses the raw signal, its first derivative, and differential curves, which show the differences in raw signals and first derivatives between the sample and the control (Figure 2).

To validate the 1D-CNN architecture, its performance was compared against three classical machine-learning algorithms: Random Forest, XGBoost, and LightGBM. The evaluation was conducted using data from two different PCR thermocyclers DTprime and CFX96 to assess robustness across laboratory equipment. Classical models utilized the same 11-channel feature vectors as the 1D-CNN.

All investigated algorithms demonstrated absolute accuracy on the DTprime thermocycler. However, validation on the CFX96 platform revealed a slight decrease in the performance of tree-based models (Table 4). Detailed classification reports indicated that errors were primarily concentrated in the discrimination of the genetically similar 7TA and 8TA alleles, specifically mistaking the 7TA/8TA heterozygote for 7TA/7TA or 8TA/8TA homozygotes (the detailed per-class breakdown is provided in Supplementary Table S1). In contrast, the 1D-CNN ensemble demonstrated absolute cross-platform robustness. On the internal validation dataset (n = 325 model specimens), the neural network maintained 100% accuracy across all metrics, successfully differentiating the complex 7TA/7TA, 7TA/8TA, and 8TA/8TA clusters without error.

**TABLE 4:** Comparative performance of classification algorithms across different PCR thermocyclers.

Algorithm	Accuracy		Macro F1-score	
	DTprime	CFX96	DTprime	CFX96
1D-CNN	100%	100%	1.00	1.00
Random forest	100%	99.1%	1.00	0.99
XGBoost	100%	99.1%	1.00	0.99
LightGBM	100%	99.47%	1.00	0.99

To evaluate the clinical generalizability of the trained 1D-CNN ensemble and to exclude optimistic bias or data leakage associated with the synthetic training data, the final model was applied to an independent external cohort of 440 clinical genomic DNA specimens, withheld entirely from architecture selection, hyperparameter optimization, and training. As noted in Section 2.1, this diagnostic cohort over-represents reduced-function and rare genotypes as a consequence of the clinical indication and therefore contains a markedly higher proportion of reduced-function and rare genotypes than the general population; while this makes it unsuitable for frequency estimation, it constitutes a demanding test of the genotype boundaries of greatest analytical concern.

On this external set, the automated 1D-CNN calls agreed with the reference pyrosequencing genotype in 440 of 440 specimens (100%; 95% one-sided lower confidence bound 99.3%, Clopper-Pearson). Agreement was complete across all seven genotype classes present in the cohort, including the 21 native 7TA/8TA specimens that represent the assay’s principal discrimination challenge and that were systematically misclassified by the tree-based models (Table 5). Two limits on the strength of this result should be stated explicitly. First, per-class confidence intervals for the rarest observed genotypes are necessarily wide: the 7TA/8TA estimate carries a lower bound of 86.7% on n = 21 - so complete agreement constitutes strong but not exhaustive evidence for these classes. Second, three genotype classes (5TA/5TA, 5TA/8TA, and homozygous 8TA/8TA) did not occur in the clinical cohort, consistent with their rarity in this population; performance on these classes was established on the engineered plasmid constructs (Table 1) and has not been independently confirmed on patient-derived material.

**TABLE 5:** Concordance analysis of automated 1D-CNN genotype calls against clinically validated reference pyrosequencing.

Genotype	Number of samples	Concordance status with references genotype	Percent agreement (95% clopper-pearson CI)
5TA/6TA	7	7/7	100% (65.18%–100%)
5TA/7TA	9	9/9	100% (71.69%–100%)
6TA/6TA	130	130/130	100% (97.72%–100%)
6TA/7TA	130	130/130	100% (97.72%–100%)
7TA/7TA	140	140/140	100% (97.88%–100%)
6TA/8TA	3	3/3	100% (36.84%–100%)
7TA/8TA	21	21/21	100% (86.74%–100%)
Total	440	440/440	100% (99.32%–100%)

The results were independently verified using two distinct PCR, devices (the CFX96 and the DTprime).

The analytical sensitivity (Limit of Detection, LOD) of the assay was evaluated using serial dilutions of the model plasmid constructs. While the absolute analytical LOD was determined to be 5 × 10^3^ copies/mL, at this low concentration (corresponding to approximately 500 copies per reaction), stochastic amplification effects increase the risk of allele dropout, potentially leading to the misclassification of a heterozygous sample as a homozygote. Consequently, to ensure absolute diagnostic reliability for biallelic genotyping, the practical LOD was strictly established at 10^4^ copies/mL. Intra-run and inter-run reproducibility were fully confirmed by the 100% cross-platform concordance of the dual-control 1D-CNN pipeline.

### 3.3 Analysis of the frequency distribution of rs3064744 alleles

This study explored the global distribution of rs3064744 alleles and genotypes by analyzing whole-genome sequencing data from representatives of five macro-populations, drawing on resources from the 1000 Genomes Project. The obtained results demonstrate ethnospecificity and a high degree of genetic heterogeneity at the studied locus. Detailed information on the distribution of alleles and genotypes among various populations is provided in Table 6; Supplementary Table S2.

**TABLE 6:** Allele frequencies of the rs3064744 polymorphism in the *UGT1A1* gene across global 1,000 Genomes populations (whole-genome sequencing data) and external reference databases (%).

Project	Population	Alleles				Comparison with EUR (1,000 genomes) population (*p*-value)***
		5TA	6TA	7TA	8TA	
1,000 genomes	AFR (n = 659)	9.9	40.3	45.1	4.7	<0.001
	AMR (n = 346)	0.7	61.3	37.7	0.3	<0.001
	EAS (n = 503)	0.0	86.7	13.3	0.0	<0.001
	EUR (n = 502)	0.4	69.9	29.7	0.0	–
	SAS (n = 488)	0.1	55.8	44.0	0.1	<0.001
gnomAD v4.1.1	EUR (n = 564,271)*	0.3	≈69.3**	30.3	0.1	0.9
FMBA database	RUS (n = 120,682)*	0.3	≈63.8**	35.9	0.06	<0.001

* For aggregated repositories (gnomAD, and FMBA), sample sizes (n) are indicative of the number of alleles analyzed divided by two.

** For the gnomAD, and FMBA, databases, the frequency of the wild-type 6TA, allele was calculated as the residual percentage, defined as 100% minus the sum of the frequencies of the alleles 5TA, 7TA, and 8TA.

*** Rare alleles (5TA, and 8TA) were excluded from the primary statistical analysis (χ2 test) to avoid potential biases associated with low cell counts.

To assess the potential impact of methodological approaches on population frequency estimates, we compared our findings with large-scale sequencing databases, specifically gnomAD v4.1.1 for the European population and the FMBA Genome Database for the Russian population (Table 6). While the gnomAD EUR frequencies closely mirrored the 1,000 Genomes EUR data generated via the HipSTR pipeline (30.3% vs. 29.7% for the 7TA allele, p = 0.9), the data from the FMBA database exhibited a noticeable deviation. Specifically, the FMBA database reported a considerably higher frequency of the clinically significant 7TA allele (35.9%) and a correspondingly lower frequency of the wild-type 6TA allele (63.8%). This distribution significantly differs (p < 0.001) from both the standard European datasets and the combined frequencies obtained from our regional cohorts using aPCR-MCA method.

The African population showcases the highest genetic diversity. It is unique among macro-groups in that the 7TA allele dominates over the wild-type 6TA allele. Additionally, the rarest minor alleles, 5TA and 8TA, are most frequently found here. Examining specific African ethnic groups and descendants reveals an exceptional distribution of genotypes. Complex heterozygotes featuring rare alleles, such as 5TA/6TA, 5TA/7TA, 6TA/8TA, and 7TA/8TA, are prevalent. Notably, the rare homozygous 5TA/5TA genotype reaches up to 3% in certain West African populations, like the Esan people. The classical 6TA/6TA genotype’s frequency drops to critically low levels in some East African groups, such as the Luhya population (9.1%). Meanwhile, the proportion of 7TA/7TA homozygotes peaks globally, with up to 27.8% among the Yoruba population.

East Asians demonstrate a high degree of genetic homogeneity. The vast majority possess the 6TA allele, with the 7TA allele being present at the lowest frequency among all populations. The alleles 5TA and 8TA are entirely absent. The majority of East Asians are homozygous for the 6TA/6TA genotype, with frequencies ranging from 70.2% in Japanese individuals to 81.8% in Vietnamese individuals. Conversely, the 7TA/7TA genotype, which is associated with GS, is extremely rare, occurring in only 0%–5.8% of the population.

The European population exhibits an intermediate distribution with a notable prevalence of the wild-type 6TA allele. The occurrence of the clinically significant 7TA allele does not exceed 30%. The rare 5TA allele is present at a frequency of less than 0.5%, and the 8TA allele is not detected within the population. In various European cohorts, the 6TA/6TA genotype constitutes the most frequent genotype, ranging from 42.4% to 54.9%, which is comparable to the distribution observed in East Asian populations. However, the proportion of 6TA/7TA heterozygotes is significantly higher, varying from 36.3% to 45.5%. The 7TA/7TA homozygote frequency falls within the range of 7.5%–13.1%.

Populations of South Asian and mixed American ancestry exhibit complex allele distribution patterns. In both groups, the 6TA allele is prevalent, while the 7TA allele maintains a consistently high frequency. Rare alleles, such as 5TA and 8TA, are present, but their cumulative frequency does not exceed 1%. Samples from these regions are notable for a high proportion of 6TA/7TA heterozygosity. For example, in Puerto Rican, Peruvian, and Sri Lankan populations, this genotype is observed in approximately half of the individuals (47%, 54%, and 56%, respectively).

To establish a more robust reference framework beyond the 1000 Genomes project, the allele frequencies of the pan-European population from the gnomAD v4.1.1 database and the aggregated Russian population from the FMBA Genome Database were statistically evaluated against the 1,000 Genomes EUR cohort (Table 6). The gnomAD EUR dataset exhibited consistency with the 1,000 Genomes EUR baseline (p > 0.05). Conversely, a highly significant divergence was observed for the national reference repository; the FMBA database demonstrated a pronounced structural shift (p < 0.001) compared to the 1,000 Genomes EUR baseline, characterized by a lower frequency of the wild-type 6TA allele and a substantially higher prevalence of the clinically significant 7TA variant.

To expand the understanding of the population-specific distribution, an analysis of the rs3064744 alleles was performed on cohorts from four regions of the Russian Federation: the Republic of Dagestan, Moscow and Moscow Region, the Republic of Sakha (Yakutia), and Rostov Region. The genotyping procedure was carried out according to the developed methodology, and the results were systematized in Tables 7, 8.

**TABLE 7:** Distribution of genotypes rs3064744 in the Russian populations (%).

Region	5TA/6TA	6TA/6TA	6TA/7TA	7TA/7TA	7TA/8TA
Republic of Dagestan (n = 202)	0	49.5	42.6	7.9	0
Moscow and Moscow region (n = 369)	0.3	38.8	47.7	12.9	0.3
Republic of Sakha (Yakutia) (n = 309)	0.3	51.0	40	8.7	0
Rostov region (n = 114)	0	50.7	40.1	9.2	0

The rare genotypes 5TA/5TA, 5TA/7TA, 5TA/8TA, 6TA/8TA, and 8TA/8TA, were not detected in any regional cohorts.

**TABLE 8:** Statistical comparison of genotypes rs3064744 distributions between Russian regional cohorts and the EUR population.

Cohort	Absolute genotype counts (6TA/6TA : 6TA/7TA : 7TA/7TA)*	Comparison with EUR population (*p*-value)
EUR (1000 Genomes)	246 : 206: 46	-
Republic of Dagestan	100 : 86: 16	0.887
Republic of Sakha (Yakutia)	158 : 124: 27	0.854
Rostov region	58 : 46: 10	0.912
Moscow and Moscow region	144 : 177: 48	<0.001

* Rare genotypes (5TA/6TA, 7TA/8TA) accounted for <1% of observations and were excluded from the primary χ2 test to ensure statistical validity.

All cohorts in this study comprise archival clinical samples with undocumented ethnicity and therefore represent regional geographical populations rather than genetically isolated ethnic groups. Specifically, given Moscow’s status as a major metropolitan hub, its observed allele frequencies may reflect higher demographic admixture compared to other regions.

The analysis of allele frequencies in the Russian populations reveals a high degree of genetic similarity to the broader European population. In all studied regions, the wild-type 6TA allele is consistently the dominant allele. In the cohorts from the Republic of Dagestan, the Republic of Sakha (Yakutia), and the Rostov Region, the frequency of the 6TA allele is approximately 71%, which closely aligns with the European average of 70%. Formal statistical testing confirms that the genotype distributions in these three regions do not differ significantly from the global European population (*p* > 0.05). The frequency of the clinically significant 7TA allele in these regions ranges from approximately 29%, which is also consistent with the European pattern. Approximately 50% of the population in these regions are carriers of the homozygous 6TA/6TA genotype. The prevalence of 6TA/7TA heterozygotes varies between 40% and 43%, and the frequency of the 7TA/7TA homozygote genotype remains between 8% and 9%, which is in accordance with classical European standards.

A unique distribution of alleles was observed in the sample from Moscow and the Moscow Region. This localized shift in genotype frequencies is statistically significant both when compared to the European reference population (*p* < 0.001) and when compared to the pooled data from the other three Russian cohorts (*p* < 0.001). In this cohort, the frequency of the 6TA allele decreased to 63%, while the proportion of the 7TA allele increased to 37%. This region is characterized by a noticeable reduction in the wild-type 6TA/6TA homozygote frequency, with an increase in the proportion of individuals carrying the 7TA allele. Consequently, the 6TA/7TA heterozygous genotype becomes the predominant genotype in this region, with a frequency of 48%. The frequency of the 7TA/7TA homozygous genotype reaches the highest level among all studied Russian cohorts in this region.

Rare alleles, such as 5TA and 8TA, were found to be exceptionally infrequent in the Russian population. However, their distribution was not uniform. These alleles were completely absent in the Republic of Dagestan and the Rostov Region. In the Republic of Sakha (Yakutia), isolated cases of the 5TA/6TA heterozygous genotype were recorded, with a frequency of 0.3%. The Moscow and Moscow Region cohort demonstrated the greatest diversity of rare variants, with both the 5TA/6TA (0.3%) and complex 7TA/8TA (0.3%) heterozygous genotypes detected.

**FIGURE 3:**
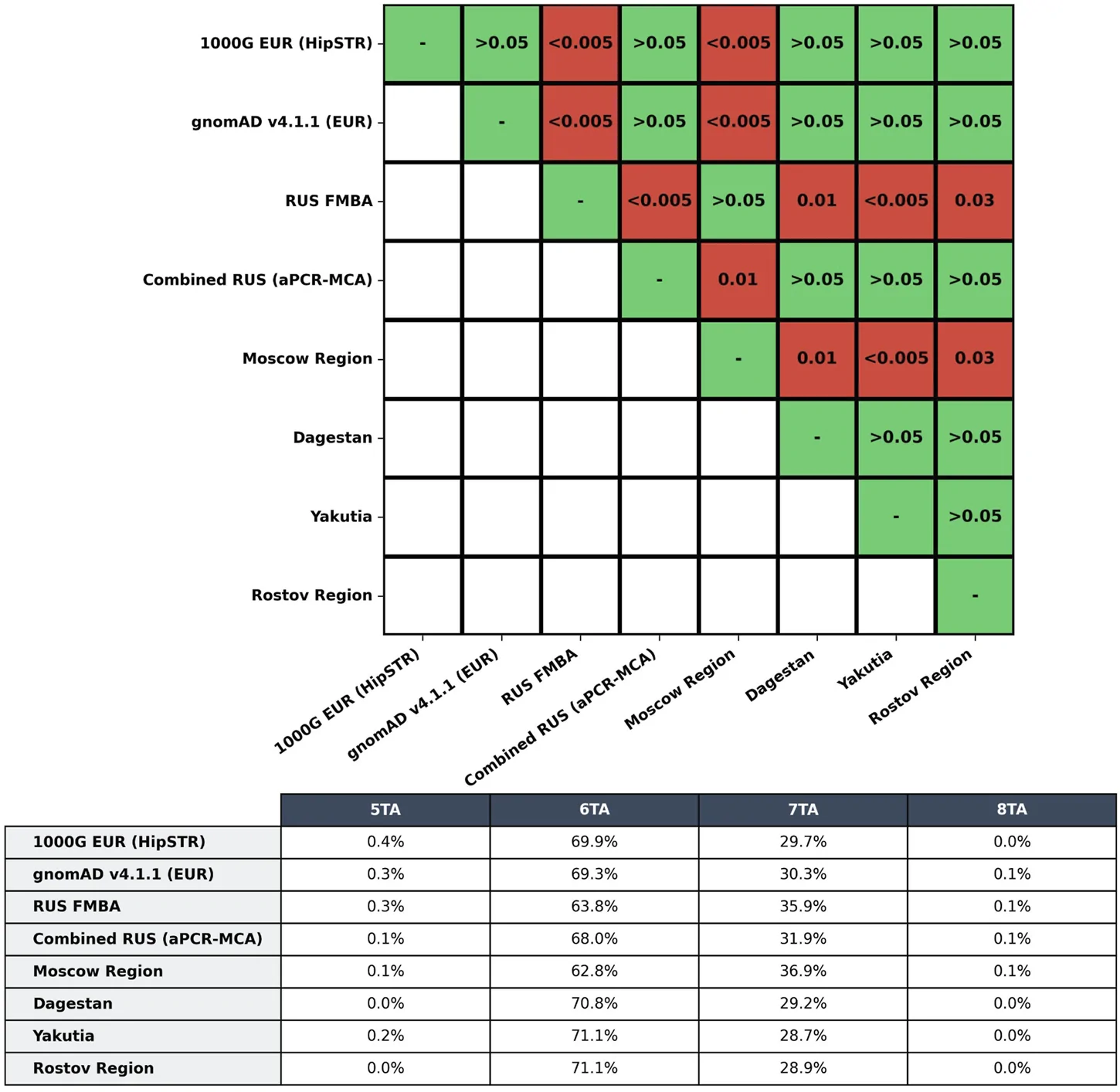
Pairwise comparative matrix and allele frequency distribution of the rs3064744 across the evaluated cohorts. Note. The upper triangular matrix displays pairwise comparisons calculated using χ^2^ test of independence, with exact p-values indicated within each cell. Green cells represent the absence of statistically significant differences (p > 0.05), while red cells highlight significant genetic divergences (p < 0.05). The corresponding allele frequencies (%) for each evaluated population compared to reference database are summarized in the integrated table below the comparative matrix.

To systematically evaluate the statistical significance of the observed allele frequency differences across all analyzed cohorts and reference databases, a pairwise comparison matrix was constructed using the χ^2^ test (Figure 3).

The heatmap visually summarizes the degree of genetic divergence. Notably, the combined Russian cohort analyzed by our aPCR-MCA method demonstrated no statistically significant differences (p > 0.05) in allele distribution when compared to the 1,000 Genomes EUR population and the gnomAD (EUR) database. However, significant structural discrepancies (p < 0.005) were revealed when comparing the international European databases and our combined aPCR-MCA cohort against the aggregated Russian data from the FMBA genome database. Further, within the regional analysis, the Moscow Region cohort formed a distinct cluster, showing statistically significant divergence (p < 0.05) from the other Russian regions (Dagestan, Yakutia, Rostov Region) and the pan-European references, but without statistically significant differences when compared with the FMBA genome database.

## 4 Discussion

The accurate genotyping of the *UGT1A1* rs3064744 polymorphism is critical for both the diagnosis of GS and pharmacogenetic testing before administering potentially toxic therapies like irinotecan or atazanavir (Raijmakers et al., 2000; Ibrahim et al., 2024; Loriot et al., 2024; Tuteja et al., 2025). Compared to alternative methods such as DNA sequencing, PCR-based approaches demonstrate superior practicality for routine clinical genotyping of the *UGT1A1* rs3064744 polymorphism, as they combine high reliability with significantly lower costs, faster turnaround times, and broader accessibility in standard molecular diagnostic laboratories. However, the extreme AT-rich nature of this promoter region renders standard diagnostic approaches, such as traditional TaqMan probes, highly inefficient (Daprà et al., 2021). This limitation is primarily due to the reduced efficiency of probe annealing at lower temperatures and the compromised discriminatory capability of these probes. In this study, we successfully overcame these limitations by developing an optimized aPCR-MCA using LNA-modified probes, achieving distinct and reproducible resolution of all four alleles (5TA, 6TA, 7TA, and 8TA). The operational advantages of this proposed approach compared to standard methodologies are summarized in Table 9.

**TABLE 9:** Comparative operational characteristics of the rs3064744 genotyping methodologies (Sissung et al., 2020; Bravo-Gómez et al., 2022).

Characteristic	Proposed aPCR-MCA	Sanger sequencing	Fragment analysis	Pyrosequencing
Turnaround time	≈2.5 h	1–2 days	1–2 days	4–6 h
Relative reagent cost	Low	High	Medium/High	High
Required equipment	1 instrument (real-time PCR)	2 instruments (thermocycler + capillary sequencer)	2 instruments (thermocycler + capillary sequencer)	2 instruments (thermocycler + pyrosequencer)
Throughput	High (96–384 well)	Low/Medium	Medium	Medium (24 well)
Automated interpretation	Yes	No (manual review)	No (manual review)	Semi-automated
Susceptibility to PCR stutter	None	None	High	None

A major strength and key technical innovation of our developed method lies in its ability to resolve all 10 genotypes (including rare variants like 8TA) within a single reaction tube using a single fluorescent probe. Consequently, the remaining 3–5 available fluorescent channels can be allocated to concurrently identify other critical genetic polymorphisms in a multiplex format. This open architecture provides a direct technical pathway for the future integration of additional clinically important variations, such as the UGT1A1*6 (rs4148323) and UGT1A1*27 (rs35350960) alleles, thereby expanding the assay into a comprehensive pharmacogenetic panel suited for multi-ethnic populations. For diagnostic laboratories, particularly those in regional or remote settings without access to costly capillary sequencers, pyrosequencing platforms, or highly specialized bioinformatics personnel, this framework offers an accessible, high-throughput, and clinically applicable solution for pharmacogenomics. Previously, methods based on various modifications of the MCA method had already been used, but either only for the 6TA and 7TA alleles or had low resolution (Farrar et al., 2011; Minucci et al., 2010). A key advancement of our method lies in the successful detection of all 10 genotypes within a single probe and a single reaction tube.

Another pivotal achievement of this work is the application of a deep learning pipeline to automate STR genotyping from post-PCR data, which entirely eliminates the subjectivity of manual interpretation. While our evaluation showed that classical tree-based machine learning algorithms (such as Random Forest, XGBoost, and LightGBM) performed well on the primary training thermocycler DTprime, they exhibited a slight performance degradation when validated on an alternative platform CFX96, specifically in differentiating the highly homologous and physically overlapping 7TA and 8TA alleles. In contrast, our ensemble of one-dimensional Convolutional Neural Networks (1D-CNNs) demonstrated consistent cross-platform accuracy. The efficacy of one-dimensional convolutional layers in extracting hierarchical spatial features from curve morphology, including the analysis of first and second derivatives alongside control parameters, proved to be a superior strategy compared to the use of flattened feature vectors within gradient boosting algorithms, as previously demonstrated (Ozkok and Celik, 2022). These results compellingly demonstrate that the 1D-CNN architecture, featuring a dual control sample comparison mechanism, represents a universal, robust, and scalable solution, independent of the specific optical and thermodynamic characteristics of the PCR thermocycler. Furthermore, although absolute Tm can exhibit minor variations between different thermocycler instruments due to distinct optical configurations and block heating dynamics, any such thermal shift occurs systematically and uniformly for all reaction wells within a single PCR run. Consequently, the integration of internal reference anchors (7TA/7TA and 7TA/8TA) within our dual control sample comparison framework effectively neutralizes this instrument-specific effect. By training the 1D-CNN to evaluate relative morphological divergence rather than absolute Tm thresholds, the assay mitigates inter-instrument variance and ensures high cross-platform robustness. This approach can also be applied to other MCA techniques, including those targeting a broader range of polymorphisms.

Applying this robust methodology, we conducted a comprehensive epidemiological assessment of the rs3064744 distribution in Russian population. Within the Russian Federation, the cohorts from the Republic of Dagestan, the Republic of Sakha (Yakutia), and the Rostov Region exhibited a genetic profile highly consistent with the broader European population, characterized by a 70% frequency of the wild-type 6TA allele. However, the Moscow and Moscow Region cohort presented a notable anomaly: the frequency of the clinically significant 7TA allele was significantly elevated to 36.9%. Given that the screening used retrospective archival samples without documented ethnicity, this localized genetic shift may reflect the profound multi-ethnic admixture and intensive internal migration flows inherent to the Moscow metropolis. This elevated frequency is corroborated by the aggregated data from the national FMBA Genome Database (35.9%), with no statistically significant difference between the two datasets (p = 0.55). While this concordance might partially reflect a metropolitan-centric sample distribution within the federal repository, it also underscores the need to interpret these frequencies in light of methodological limitations inherent to large-scale sequencing databases. Notably, the absence of genealogical data in the source archival collection precludes definitive conclusions about population structure; thus, the observed trend remains hypothetical and requires validation in prospective studies with documented ethnicity.

At the same time, this elevated frequency aligns closely with the findings of several earlier studies evaluating the prevalence of the rs3064744 in Russian cohorts (Ivanova et al., 2023; Ivanov and Semenova, 2021; Volkov and Tsurkan, 2019). This concordance suggests that these previous datasets may either share a similar clinical ascertainment bias or, alternatively, reflect the true underlying allele frequency within the broader population.

As highlighted by our comparative analysis, there is a statistically significant structural discrepancy (p < 0.005) between the regional allele frequencies obtained via our targeted aPCR-MCA method and the aggregated data from the FMBA database. This discrepancy perfectly illustrates the inherent vulnerability of conventional short-read sequencing pipelines utilized in large-scale repositories like gnomAD, ALFA, and FMBA when genotyping highly polymorphic STRs. The UGT1A1 promoter’s extreme AT-rich sequence is highly susceptible to polymerase slippage during NGS library preparation, generating prominent artifacts. Standard short-read alignment algorithms frequently misinterpret this noise, leading to a systematic overestimation of expanded alleles (such as 7TA) and a corresponding underestimation of the wild-type 6TA sequence. This bioinformatic limitation is further evidenced by the presence of biologically anomalous structural variants (e.g., abnormally contracted 4TA or hyper-expanded 10TA and 11TA, along with fragmented indels) frequently registered in the raw variant logs of gnomAD. These anomalies are widely recognized in the literature as algorithmic alignment artifacts associated with short-read sequencing limitations (Sissung et al., 2020; Willems et al., 2017), rather than true physiological alleles recognized by official nomenclature (UGT1A1 allele nomenclature, 2015). Therefore, the observed statistical discrepancies provide compelling evidence that standard short-read WGS pipelines may not accurately capture the true population architecture of the rs3064744, thereby confirming the methodological advantage and necessity of STR-specific PCR approaches for precise clinical genotyping.

Importantly, our screening successfully identified rare alleles, including the 5TA/6TA and complex 7TA/8TA genotypes, in the Yakutia and Moscow cohorts. The reliable identification of these minor alleles, which frequently confound traditional software, underscores the critical clinical utility of combining aPCR-MCA with advanced deep learning interpretation for complex STR genotyping. By automating interpretation and eliminating human operator bias, this integrated system guarantees reproducible and reliable pharmacogenetic genotyping, even for the most challenging alleles.

Our research encountered several limitations. Firstly, the lack of phenotypic and clinical data restricted the depth of our investigation. Secondly, while the geographic scope of our population screening encompassed four geographically and culturally distinct regions of the Russian Federation (Moscow, Dagestan, Yakutia, and the Rostov Region), the findings cannot be fully extrapolated to the entire multi-ethnic population of the country. This underscores the necessity for future screening initiatives to be expanded to additional regions across Siberia, the Urals, and the Far East.

Finally, while the proposed aPCR-MCA and 1D-CNN pipeline demonstrates robust analytical performance and high clinical concordance, formal validation in accordance with local and international regulatory frameworks for *in-vitro* diagnostic medical devices would be a prerequisite prior to its deployment as a standalone clinical diagnostic tool.

## Data Availability

The complete source code for the model training process is publicly accessible via the following hyperlink: https://github.com/michaelvinockurov/NNGilbert.
