# Supplementary material for: Development of a PCR-based technique for genotyping UGT1A1 gene and distribution of rs3064744 alleles in the Russian population
Source: Front Genet. 2026 Aug 26;17:1899437. doi: 10.3389/fgene.2026.1899437 (PMC13553059; doi:10.3389/fgene.2026.1899437)
Supplement: Supplementary file 1 [file Table2.docx]

Table S1. Genotype frequencies of the rs3064744 polymorphism in the *UGT1A1* gene based on 1000 Genomes Project whole-genome sequencing data.

| Population | Subpopulation* | 5/5 | 5/6 | 5/7 | 5/8 | 6/6 | 6/7 | 6/8 | 7/7 | 7/8 | 8/8 | n |
| --- | --- | --- | --- | --- | --- | --- | --- | --- | --- | --- | --- | --- |
| AFR | ACB |  | 9.4% | 8.3% |  | 20.8% | 29.2% | 4.2% | 22.9% | 5.2% |  | 96 |
|  | ASW |  | 6.6% | 8.2% |  | 18% | 44.3% | 4.9% | 14.8% | 3.3% |  | 61 |
|  | ESN | 3.1% | 6.1% | 15.3% | 3.1% | 16.3% | 35.7% | 2% | 17.3% | 1% |  | 98 |
|  | GWD |  | 9.7% | 10.6% | 0.9% | 14.2% | 37.2% | 5.3% | 15% | 7.1% |  | 113 |
|  | LWK |  | 6.1% | 11.1% |  | 9.1% | 47.5% | 4% | 18.2% | 3% | 1% | 99 |
|  | MSL |  | 2.4% | 8.3% | 1.2% | 21.4% | 40.5% | 3.6% | 14.3% | 8.3% |  | 84 |
|  | YRI | 0.9% | 9.3% | 9.3% | 0.9% | 15.7% | 30.6% | 0.9% | 27.8% | 4.6% |  | 108 |
| AMR | CLM |  |  |  |  | 45.7% | 41.5% |  | 12.8% |  |  | 94 |
|  | MXL | 1.6% |  |  |  | 37.5% | 48.4% |  | 10.9% | 1.6% |  | 64 |
|  | PEL |  | 1.2% |  |  | 26.2% | 53.6% |  | 19% |  |  | 84 |
|  | PUR |  | 1.9% |  |  | 37.5% | 47.1% | 1% | 12.5% |  |  | 104 |
| EAS | CDX |  |  |  |  | 72% | 24.7% |  | 3.2% |  |  | 93 |
|  | CHB |  |  |  |  | 77.5% | 20.6% |  | 2% |  |  | 102 |
|  | CHS |  |  |  |  | 78.1% | 20% |  | 1.9% |  |  | 105 |
|  | JPT |  |  |  |  | 70.2% | 24% |  | 5.8% |  |  | 104 |
|  | KHV |  |  |  |  | 81.8% | 18.2% |  | 0% |  |  | 99 |
| EUR | CEU |  |  |  |  | 46.5% | 45.5% |  | 8.1% |  |  | 99 |
|  | FIN |  |  |  |  | 42.4% | 44.4% |  | 13.1% |  |  | 99 |
|  | GBR |  |  |  |  | 54.9% | 36.3% |  | 8.8% |  |  | 91 |
|  | IBS |  | 0.9% |  |  | 50.9% | 39.6% |  | 8.5% |  |  | 106 |
|  | TSI |  | 2.8% |  |  | 50.5% | 39.3% |  | 7.5% |  |  | 107 |
| SAS | BEB |  |  |  |  | 29.1% | 55.8% |  | 15.1% |  |  | 86 |
|  | GIH |  |  |  |  | 35% | 40.8% |  | 24.3% |  |  | 103 |
|  | ITU |  |  |  |  | 37.6% | 41.6% |  | 20.8% |  |  | 101 |
|  | PJL |  | 1% |  |  | 28.1% | 53.1% |  | 16.7% | 1% |  | 96 |
|  | STU |  |  |  |  | 25.5% | 55.9% |  | 18.6% |  |  | 102 |

**ACB – African Caribbean in Barbados; ASW – Americans of African Ancestry in SW USA; ESN – Esan in Nigeria; GWD – Guinea‑Bissau; LWK – Luhya in Webuye, Kenya; MSL – Mende in Sierra Leone; YRI – Yoruba in Ibadan, Nigeria; CLM – Colombian from Medellin, Colombia; MXL – Mexican Ancestry from Los Angeles, USA; PEL – Peruvian from Lima, Peru; PUR – Puerto Rican from Puerto Rico; CDX – Chinese Dai in Xishuangbanna, China; CHB – Han Chinese in Beijing, China; CHS – Southern Han Chinese; JPT – Japanese in Tokyo, Japan; KHV – Kinh in Ho Chi Minh City, Vietnam; CEU – Utah Residents (CEPH) with Northern and Western European ancestry; FIN – Finnish in Finland; GBR – British in England and Scotland; IBS – Iberian population in Spain; TSI – Toscani in Italy; BEB – Bengali from Bangladesh; GIH – Gujarati Indian from Houston, Texas; ITU – Indian Telugu from the UK; PJL – Punjabi from Lahore, Pakistan; STU – Sri Lankan Tamil from the UK.*
